# Die psychische Belastung von Kindern, Jugendlichen und ihren Familien während der COVID-19-Pandemie und der Zusammenhang mit emotionalen und Verhaltensauffälligkeiten

**DOI:** 10.1007/s00103-021-03455-1

**Published:** 2021-11-09

**Authors:** Manfred Döpfner, Julia Adam, Carolina Habbel, Birte Schulte, Karen Schulze-Husmann, Michael Simons, Fabiola Heuer, Christiane Wegner, Stephan Bender, TEMPO-Studiengruppe, Manfred Döpfner, Julia Adam, Carolina Habbel, Karen Schulze-Husmann, Michael Simons, Jan Schwendowius, Fabiola Heuer, Christiane Wegner, Stephan Bender, Beate Herpertz-Dahlmann, Luise Poustka, B-FAST-Studiengruppe, Birte Schulte, Stephan Bender, Juliane Münch, Burkhard Tönshoff, Alexander Joachim, Lena T. Birzele, Eva Möhler

**Affiliations:** 1 Ausbildungsinstitut für Kinder- und Jugendlichenpsychotherapie (AKiP), Medizinische Fakultät und Uniklinik Köln, Universität zu Köln, Köln, Deutschland; 2 Klinik für Psychiatrie, Psychosomatik und Psychotherapie des Kindes- und Jugendalters, Medizinische Fakultät und Uniklinik Köln, Universität zu Köln, Robert-Koch-Str. 10, 50931 Köln, Deutschland; 3 Klinik für Psychiatrie, Psychosomatik und Psychotherapie des Kindes- und Jugendalters, RWTH Aachen, Aachen, Deutschland; 4 Klinik für Psychiatrie, Psychosomatik und Psychotherapie des Kindes- und Jugendalters, Charité, Universitätsmedizin Berlin, Berlin, Deutschland; 5 Klinik für Kinder- und Jugendpsychiatrie/Psychotherapie, Universitätsmedizin Göttingen, Georg-August-Universität Göttingen, Göttingen, Deutschland

**Keywords:** COVID-19, Kinder und Jugendliche, Psychische Belastung, Verhaltensauffälligkeiten, Emotionale Auffälligkeiten, COVID-19, Children and adolescents, Mental burden, Behavior problems, Emotional problems

## Abstract

**Hintergrund und Ziel:**

Die im Rahmen der COVID-19-Pandemie erlassenen Maßnahmen zum Infektionsschutz führten zu tiefgreifenden Einschränkungen und Veränderungen im sozialen, (vor-)schulischen, familiären und Freizeitbereich. Die vorliegende Studie untersucht das Ausmaß an psychischer Belastung von Kindern, Jugendlichen und ihren Familien während der COVID-19-Pandemie. Mögliche Einflussfaktoren sollen identifiziert werden.

**Material und Methoden:**

Die Untersuchungen erfolgten zwischen Herbst 2020 und Frühjahr 2021 in einer klinischen Inanspruchnahmestichprobe (*n* = 280 Patient:innen zwischen 4–17 Jahren) und einer Feldstichprobe (*n* = 1958 Kinder und Jugendliche zwischen 4–19 Jahren, über Schulen und vorschulische Einrichtungen rekrutiert). Dabei wurden Urteile der Eltern sowie Selbsturteile der Kinder und Jugendlichen mittels Fragebögen erfasst.

**Ergebnisse:**

Die psychische Belastung der Kinder und Jugendlichen im Zusammenhang mit der Pandemie wird über beide Beurteilungsperspektiven und Stichproben hinweg als leicht bis moderat erhöht eingeschätzt. Rund 60–70 % der Eltern- und Selbsturteile beschreiben eine Zunahme dieser Belastung, während Entlastungen von bis zu 12 % sowohl im Eltern- als auch im Selbsturteil angegeben werden. Beim Vergleich der beiden Stichproben zeigt sich eine leicht höhere Belastung der Kinder und Jugendlichen nur im Selbsturteil der *Klinikstichprobe*. Die untersuchten soziodemografischen Faktoren haben keinen Einfluss auf die Belastung. Allerdings zeigen sich in beiden Stichproben leichte bis moderate Zusammenhänge zwischen der subjektiv erlebten Verschlechterung der familiären und sozialen Situation und einem erhöhten Belastungserleben.

**Diskussion:**

Während einer Pandemie sollten gezielte Interventionen für belastete Subgruppen angeboten werden. Universelle Interventionen sind nicht indiziert.

**Zusatzmaterial online:**

Zusätzliche Informationen sind in der Online-Version dieses Artikels (10.1007/s00103-021-03455-1) enthalten.

## Abstract (en)

**Background and objective:**

The infection protection measures adopted as part of the COVID-19 pandemic led to profound restrictions and changes in the social, (pre-) school, family, and leisure areas. The objective of the current study was to examine the mental burden of children and adolescents and their families during the COVID-19 pandemic. Furthermore, this study aimed to identify possible factors that influence the mental burden.

**Materials and methods:**

The examinations were carried out between autumn 2020 and spring 2021 in a clinical sample (*n* = 280 patients aged 4–17 years) and a community sample (*n* = 1958 children and adolescents aged 4–19 years recruited via schools and preschools). Ratings of parents as well as children and adolescents via questionnaires were assessed.

**Results:**

Mental burden due to the corona pandemic was assessed as slightly to moderately increased across both rating perspectives and both samples. Overall, around 60 to 70% of the parents, children, and adolescents describe an increase in mental burden; in contrast, up to 12% of parents as well as children and adolescents describe relief. When comparing both samples, a slightly higher burden on children and adolescents can only be seen in the self-assessment of the *clinical sample*. None of the socio-demographic factors analyzed influences the mental burden statistically significant. However, low to moderate correlations between the subjectively experienced deterioration in the family and social situation and an increased level of stress is found.

**Discussion:**

Targeted interventions for exposed subgroups should be offered during a pandemic. Universal interventions are not indicated.

## Einleitung

Die COVID-19-Pandemie und die damit einhergegangenen Infektionsschutzmaßnahmen, die sowohl Kinder als auch Eltern betrafen (v. a. Quarantänemaßnahmen in Schulen, Kontakteinschränkungen, vermehrte Homeofficetätigkeiten), führten zu tiefgreifenden Veränderungen des täglichen Lebens von Kindern, Jugendlichen und ihren Familien. Dadurch wurden die Kinder, Jugendlichen und ihre Familien erheblichen psychischen Belastungen ausgesetzt [1, 2].

Die aktuelle empirische Literatur zeigt, dass – je nach Operationalisierung und Untersuchungsgruppe – 15–71 % der Kinder und Jugendlichen [3–5] sowie 30–75 % der Bezugspersonen erhöhte Belastungen schildern [4, 6, 7]. Einige Studien zeigen erhöhte Raten von Depressionen und Angstsymptomen sowohl bei Kindern als auch bei Bezugspersonen [6, 8–10], teilweise auch erhöhte Hyperaktivität sowie vermehrte Unaufmerksamkeit und oppositionelles Verhalten [4, 11]. In der ersten deutschlandweiten repräsentativen Studie, der COPSY-Studie [12, 13], gaben 71 % der Kinder und Jugendlichen und 75 % der Eltern an, dass sie sich durch die erste Welle der Pandemie belastet fühlten. Im Vergleich zu den Ergebnissen vor der Pandemie war die Lebensqualität der Kinder bedeutsam reduziert und die Rate der Kinder mit psychischen Auffälligkeiten verdoppelte sich annähernd von 9,9 % auf 17,8 %.

Die Vermutung liegt nahe, dass durch die COVID-19-Pandemie psychische Belastungen und Störungen bei bereits psychisch auffälligen Kindern und Jugendlichen stärker ausgeprägt sind als bei psychisch unauffälligen. Einige Studien konnten erhöhte Raten von psychischen Belastungen und Störungen bei Kindern mit vorbestehenden psychischen Auffälligkeiten nachweisen [11, 14], andere nicht [15–17].

Eine Erfassung von psychischen Belastungen und Störungen sowohl im Selbsturteil von Kindern und Jugendlichen als auch im Elternurteil ist deshalb besonders aufschlussreich, weil das Selbsturteil erheblich vom Urteil der Eltern differieren kann [18, 19]. Allerdings liegt unseres Wissens bislang nur eine Studie vor, die Selbst- und Elternurteil zu Belastungen und psychischen Auffälligkeiten von Kindern und Jugendlichen mit und ohne psychische Störungen während der Coronapandemie parallel erhoben hat [20]. In dieser kanadischen Studie konnte bei rund 70 % der Kinder und Jugendlichen in zumindest einem von 6 Bereichen (Depression, Angst, Reizbarkeit, Unaufmerksamkeit, Hyperaktivität, Zwanghaftigkeit) der psychischen Gesundheit eine Verschlechterung festgestellt werden, wobei die Unterschiede zwischen Kindern und Jugendlichen mit und ohne psychische Störungen gering waren.

Neben Belastungen können die Veränderungen im Zusammenhang mit der Pandemie auch zu Entlastungen bei Kindern, Jugendlichen und ihren Familien führen [21, 22]. So kann das Aussetzen des normalen Schulbesuchs schulischen Stress verringern, die Reduktion von Gleichaltrigenkontakten kann Kinder und Jugendliche mit Kontaktproblemen zunächst entlasten. Solche Entlastungen wurden bislang zwar wesentlich seltener erhoben, Cost et al. [20] fanden jedoch bei 19–31 % der Kinder und Jugendlichen in mindestens einem Bereich eine Verbesserung der psychischen Gesundheit.

Die aktuelle Studie untersucht daher im Zeitraum Herbst 2020 bis Frühjahr 2021 in einer *Klinikstichprobe* und einer *(Vor‑)Schulstichprobe* (1) die Stärke der psychischen Belastung und der Veränderung psychischer Auffälligkeiten von Kindern und Jugendlichen während der Pandemie im Urteil der Eltern und der Kinder und Jugendlichen selbst (ab 11 Jahre) sowie (2) den Zusammenhang von psychischer Belastung mit zeitgleich erhobenen Verhaltens- und emotionalen Auffälligkeiten der Kinder und Jugendlichen im Selbst- und im Elternurteil sowie mit soziodemografischen Parametern.

## Methoden

Die Studie wurde im Rahmen des Netzwerkes Universitätsmedizin (NUM^1^), das vom Bundesministerium für Bildung und Forschung (BMBF) gefördert wird, in 2 multizentrischen Projekten durchgeführt. Die *Klinikstichprobe* wurde im Rahmen der TEMPO-Studie (Telemedizinische Psychiatrie und Psychotherapie für Kinder und Jugendliche während der COVID-19-Pandemie) (registriert im Deutschen Register Klinischer Studien, DRKS-ID: 00023525) erhoben. Die *(Vor‑)Schulstichprobe* wurde im Rahmen des Projektes B‑FAST Schulen und Kitas (Bundesweites Forschungsnetz Angewandte Surveillance und Testung) gewonnen (DRKS-ID: RKS00023911).

Die Daten wurden anhand des Datenerfassungstools REDCap (Research Electronic Data Capture) erhoben. Die Teilnehmenden füllten dabei Fragebögen über einen per E‑Mail an sie versandten Link selbst aus. Es wurden deskriptive Statistiken berechnet sowie Korrelations- und Varianzanalysen durchgeführt. T‑Tests wurden zum Vergleich von Mittelwertunterschieden zwischen Beurteilungsperspektiven (und Erhebungszeiträumen) innerhalb einer Stichprobe durchgeführt. Vergleiche zwischen den Stichproben (Feldstichprobe vs. Klinikstichprobe) erfolgten ausschließlich deskriptiv.

### Stichproben und Rekrutierung

Die *Klinikstichprobe* umfasst *n* = 280 Patient:innen zwischen 4 und 17 Jahren, die im Rahmen der Routineversorgung an kinder- und jugendpsychiatrischen Institutsambulanzen von 4 Universitätskliniken (Aachen: *n* = 7, Charité Berlin: *n* = 49, Göttingen: *n* = 40, Köln: *n* = 83) und einer Psychotherapieambulanz für Kinder und Jugendliche (AKiP Uniklinik Köln: *n* = 101) im Zeitraum vom 01.12.2020 bis 31.03.2021 (Pandemiewelle 2 bis erstes Drittel Pandemiewelle 3) vorgestellt wurden.

In die Studie wurden konsekutiv angemeldete, wiedervorgestellte bzw. weiterbehandelte Patient:innen (Alterskriterium: 4 bis 17 Jahre) eingeschlossen. Voraussetzung hierfür war die schriftliche Einwilligung der Sorgeberechtigten und Patient:innen ab 8 Jahren in die Studienteilnahme. Da das Hauptziel dieser Studie die Untersuchung der Umsetzung und Durchführung von Videosprechstunden war, wurden Patient:innen ausgeschlossen, bei denen (nach klinischem Urteil) eine Kontraindikation für eine Videosprechstunde vorlag, wie eine akute psychiatrische Notfallsituation, eine geistige Behinderung oder eine akute körperliche Symptomatik, die unmittelbar eine somatische Diagnostik bzw. Behandlung erforderte. Zudem wurden Familien mit mangelnden Deutschkenntnissen ausgeschlossen. Die Diagnosen der Klinikstichprobe basieren ebenfalls auf einem klinischen Urteil. Eine Übersicht über die Rekrutierung an den einzelnen Standorten findet sich in Tab. 1.

**Table 1:** 

	Gesamt	Aachen und Göttingen	Berlin	Köln AKiP	Köln KJP
*Ambulanztermin vereinbart: n*	1054	282	248	287	237
Erstvorstellungen	493	33	41	287	132
Wiedervorstellungen	561	249	207	0	105
*Kontraindikation: n*	447	122	160	27	138
*Indikation: n*	604	158	88	258	100
Kein Studieninteresse/Studienabbruch	324	111	39	157	17
Studieninteresse und Einwilligung	280	47	49	101	83

*AKiP* Ausbildungsinstitut für Kinder- und Jugendlichenpsychotherapie, *KJP* Kinder- und Jugendpsychiatrie, *n* absolute Häufigkeiten

Die *(Vor‑)Schulstichprobe *umfasst *n* = 1958 Kinder und Jugendliche zwischen 4 und 19 Jahren, die in Kitas oder Schulen an 5 Standorten (Düsseldorf, Heidelberg, Homburg, Köln, München) im Zeitraum vom 09.11.2020 bis zum 18.04.2021 (Anfang Pandemiewelle 2 bis Mitte Pandemiewelle 3) befragt wurden. Die teilnehmenden Einrichtungen wurden in Absprache mit den Gesundheits- und Schulämtern sowie weiteren beteiligten Institutionen ausgewählt. Die Auswahl der Einrichtungen zielte darauf ab, Regionen mit verschiedenen Bevölkerungsdichten und sozialen Kontexten abzubilden. Insgesamt waren 18 Einrichtungen (2 Kitas, 8 Grundschulen, 8 weiterführende Schulen) beteiligt. Eine Teilnahme am Projekt konnte insgesamt rund 5000 Kindern bzw. ihren Eltern angeboten werden. Eine schriftliche Einwilligung der Eltern und Schüler:innen ab 8 Jahren war Voraussetzung für eine Studienteilnahme.

### Messinstrumente

Zur Erfassung der *psychischen Belastung* der Kinder bzw. Jugendlichen und ihrer Familien durch die COVID-19-Pandemie in der *Klinikstichprobe* und der *(Vor‑)Schulstichprobe* wurde eine gekürzte Version des *Corona-Belastungsbogens* (CBB; [23]; s. Onlinematerial 1) im Elternurteil (CBB‑E; 11 Items) und im Selbsturteil (CBB-KJ; ab 11 Jahren; 7 Items) eingesetzt. Im Elternurteil wurden Veränderungen im Verhalten des Kindes oder Jugendlichen sowie Be- und Entlastungen in seinen Beziehungen und der Betreuungs-, der familiären, schulischen, beruflichen Situation erfasst wie auch vorhandene Freizeitmöglichkeiten. Die Gesamtbeurteilungen der Veränderungen für das Kind und den Elternteil selbst wurden anhand von fünfstufigen Likert-Skalen vorgenommen (−2 = „viel besser“, −1 = „etwas besser“, 0 = keine Änderung, 1 = „etwas schlechter“, 2 = „viel schlechter“). Im Selbsturteil wurden analog zum Elternurteil die gleichen Items erhoben bis auf die Beurteilung der Betreuung des Kindes, der familiären Situation insgesamt, der beruflichen Situation der Eltern und der Veränderungen für das Elternteil insgesamt. Es wurde jeweils ein Gesamtbelastungsindex über alle Items (Kennwert = Summe der Itemwerte/Anzahl der Items) gebildet, wobei ein höherer Wert eine höhere Belastung durch die Coronapandemie bedeutet. Für direkte Vergleiche zwischen CBB‑E und CBB-KJ auf Skalenebene wurde ein CBB-Cross-Informant-Index mit den jeweils im Eltern- und Selbsturteil korrespondierenden Items gebildet (7 Items). Um die subjektiv erlebten Veränderungen der familiären Rahmenbedingungen abzubilden, wurde zudem ein Index (CBB-E-Rahmenbedingungen) über die Items zu Veränderungen der Betreuungs-, beruflichen und familiären Situation sowie über die Veränderungen für die Bezugsperson selbst gebildet. Die internen Konsistenzen (Cronbachs Alpha) sind bis auf den Index CBB-E-Rahmenbedingungen (Klinikstichprobe: *α* = 0,59; (Vor‑)Schulstichprobe: *α* = 0,63) für die Gesamtindizes [und den Cross-Informant-Index] sowohl für die *Klinik-* als auch die *(Vor‑)Schulstichprobe* als ausreichend bis gut zu bewerten (CBB-E: Klinikstichprobe: *α* = 0,81 [0,79], (Vor‑)Schulstichprobe: *α* = 0,80 [0,75]; CBB-KJ: Klinikstichprobe: *α* = 0,67, (Vor‑)Schulstichprobe: *α* = 0,66).

Zur Erfassung von *emotionalen und Verhaltensauffälligkeiten* der Kinder und Jugendlichen wurden in der *Klinikstichprobe* der Elternfragebogen über das Verhalten von Kindern und Jugendlichen (Child Behavior Checklist/6–18 R [CBCL] [24]) und der Fragebogen für Jugendliche (Youth Self Report/11–18 R [YSR]; ab 11 Jahren [24]) eingesetzt.

In der *(Vor‑)Schulstichprobe* wurde zur Erfassung von emotionalen und Verhaltensauffälligkeiten der Kinder und Jugendlichen die deutschsprachige Version des *Strengths and Difficulties Questionnaire *(SDQ [25]) im Elternurteil (SDQ-E) und im Selbsturteil der Kinder und Jugendlichen ab 11 Jahren (SDQ-S) eingesetzt (s. auch [26, 27]).

Zur Beurteilung der *Funktionsbeeinträchtigungen und des Leidensdrucks der Kinder und Jugendlichen* wurden in der *Klinikstichprobe* jeweils 5 Items zur Erfassung der Beeinträchtigungen der Beziehungen, der schulischen Leistungsfähigkeit und des subjektiven Leidensdrucks (Skala: 0 = gar nicht bis 3 = besonders) aus dem Fremd- bzw. Selbstbeurteilungsbogen (ab 11 Jahren) zum *Screening psychischer Störungen* (FBB-SCREEN/SBB-SCREEN) aus dem *Diagnostik-System für psychische Störungen nach ICD-10 und DSM‑5 für Kinder und Jugendliche* (DISYPS-III [28]) eingesetzt.

Im klinischen Urteil erfolgte die *Globalbeurteilung der psychosozialen Anpassung *mithilfe der Achse 6 des *Multiaxialen Klassifikationsschemas* (MAS [29]) anhand einer 9‑stufigen Skala (0 = „Hervorragende oder gute soziale Anpassung auf allen Gebieten“ bis 8 = „Braucht ständige Betreuung [24-Stunden-Versorgung]“). Mit dem Ziel einer einfacheren Interpretierbarkeit wurde die Skala für die vorliegenden Berechnungen recodiert (0 = „Braucht ständige Betreuung [24-Stunden-Versorgung]“ bis 8 = „Hervorragende oder gute soziale Anpassung auf allen Gebieten“), sodass ein höherer Wert mit einer besseren psychosozialen Anpassung einhergeht.

Der *sozioökonomische Status* in der Klinikstichprobe wurde anhand des Berufes/der Ausbildung der Eltern erfasst, erhoben über eine *Kurzfassung der Basisdokumentation* [30]. Die daraus gebildete Skala zum sozioökonomischen Status reichte von (1) ungelernte Arbeiter:innen oder angelernte Berufe bis (5) leitende Angestellte, Beamt:innen im höheren Dienst oder Akademiker:innen, freie Berufe, größere Unternehmer:innen.

Weitere Informationen zu den Messinstrumenten sind dem Onlinematerial 2 zu entnehmen.

## Ergebnisse

### Stichprobenmerkmale

Die Merkmale der *Klinikstichprobe* insgesamt (*n* = 280) und der einzelnen Standorte sind Tab. 2 zu entnehmen (Aachen und Göttingen wurden zusammengefasst, da am Standort Aachen lediglich *n* = 7 Patient:innen eingeschlossen wurden). Über alle Standorte hinweg waren die Patient:innen im Mittel 11,75 Jahre alt (*SD* = 3,73), rund 55 % waren Jungen. In jeweils etwa einem Drittel der Fälle wurden ausschließlich externale Störungen (Aufmerksamkeitsdefizit-, Hyperaktivitätsstörung (ADHS), Störung des Sozialverhaltens) bzw. internale (Angst, Depression) oder gemischte/andere Störungen diagnostiziert. Die Ergebnisse von vergleichenden Analysen zwischen den Standorten finden sich im Onlinematerial 3.

**Table 2:** 

	Gesamt	Aachen und Göttingen	Berlin	AKiP Köln	KJP Köln
*n*	280	47	49	101	83
*Alter in Jahren: M (SD)*	11,75 (3,73)	13,18 (3,68)	10,72 (3,66)	11,08 (3,69)	12,37 (3,56)
*Männlich: n (%)*	153 (54,6)	24 (51,1)	29 (59,2)	56 (55,4)	44 (53,0)
*Wohnsituation: n (%)*					
Beide Eltern	222 (82,8)	37 (86,0)	34 (73,9)	84 (85,7)	67 (82,7)
Alleinerziehend	41 (15,3)	5 (11,6)	11 (23,9)	13 (13,3)	12 (14,8)
Andere	5 (1,9)	1 (2,3)	1 (2,2)	1 (1,0)	2 (2,5)
*Sozioökonomischer Status der Familie (nach Berufsgruppe): n (%)*					
Ungelernte Arbeiter:innen oder angelernte Berufe	21 (8,2)	5 (14,7)	6 (13,3)	6 (6,2)	4 (5,1)
Facharbeiter:innen, Handwerker:innen, Angestellte oder Beamt:innen im einfachen Dienst oder kleinste Selbstständige, ambulantes Gewerbe	53 (20,8)	10 (29,4)	7 (15,6)	19 (19,8)	17 (21,3)
Mittlere Angestellte, Beamt:innen im mittleren Dienst oder kleine selbstständige Gewerbetreibende	79 (31,0)	9 (26,5)	14 (31,1)	33 (34,4)	23 (28,7)
Höher qualifizierte Angestellte, Beamt:innen im gehobenen Dienst oder Selbstständige mit mittleren Geschäften, Betrieben	62 (24,3)	7 (20,6)	15 (33,3)	23 (24,0)	17 (21,3)
Leitende Angestellte, Beamt:innen im höheren Dienst oder Akademiker:innen, freie Berufe oder große Unternehmer:innen	40 (15,7)	3 (8,8)	3 (6,7)	15 (15,6)	19 (23,8)
*Psychosoziales Funktionsniveau*^*a*^*: M (SD)*	5,26 (1,25)	5,05 (1,19)	4,73 (1,64)	5,29 (1,07)	5,66 (1,11)
*Psychische Störungen in der Familie: n (%)*	133 (57,8)	17 (50,0)	27 (65,9)	47 (58,8)	42 (56,0)
*Somatische Erkrankungen in der Familie: n (%)*	90 (43,3)	10 (45,5)	15 (39,5)	44 (57,9)	21 (29,2)
*Diagnosen: n (%)*					
Externale Störungen	103 (36,8)	22 (46,8)	26 (53,1)	19 (18,8)	36 (43,4)
Internale Störungen	85 (30,4)	11 (23,4)	7 (14,3)	46 (45,5)	21 (25,3)
Gemischte Störungen	26 (9,3)	7 (14,9)	3 (6,1)	13 (12,9)	3 (3,6)
Andere Störungen	66 (23,6)	7 (14,9)	13 (26,5)	23 (22,8)	23 (27,7)
*Anzahl Diagnosen: n (%)*					
1 Diagnose	145 (51,8)	21 (44,7)	18 (36,7)	62 (61,4)	44 (53,0)
2 Diagnosen	99 (35,4)	15 (31,9)	22 (44,9)	34 (33,7)	28 (33,7)
3 Diagnosen	36 (12,9)	11 (23,4)	9 (18,4)	5 (5,0)	11 (13,3)

*n* absolute Häufigkeiten, *%* prozentuale Häufigkeiten, *M* Mittelwert, *SD* Standardabweichung

^a^Skala: 0 = „Braucht ständige Betreuung (24-Stunden-Versorgung)“ bis 8 = „Hervorragende oder gute soziale Anpassung auf allen Gebieten“

In Tab. 3 sind die Merkmale der *(Vor‑)Schulstichprobe* (*n* = 1024 Kinder- und Jugendlichenurteile; *n* = 1606 Elternurteile; *n* = 672 beide Urteile) dargestellt. Die im Elternurteil untersuchten Kinder waren im Mittel 10,86 Jahre alt (*SD* = 3,23), rund 45 % waren Jungen, 58 % besuchten eine weiterführende Schule. Die Schüler:innen, deren Selbsturteile vorlagen, waren im Mittel 13,87 Jahre alt (*SD* = 2,11), knapp 40 % waren Jungen. Die Ergebnisse von vergleichenden Analysen zwischen den Substichproben (sowie Erhebungszeiträumen) finden sich im Onlinematerial 3.

**Table 3:** 

	Schüler:innen	Eltern/Sorgeberechtigte	Gemeinsam beurteilt
*n*	1024	1606	672
*Alter in Jahren: M (SD)*	13,87 (2,11)	10,86 (3,23)	13,50 (1,96)
*Altersspanne in Jahren*	11–19	4–18	11–18
*Männlich: n (%)*	406 (39,6)	718 (44,7)	279 (41,5)
*Einrichtungen: n (%)*			
Kita	–	17 (1,1)	–
Grundstufe	–	652 (40,6)	–
Weiterführende Schule	1024 (100)	937 (58,3)	672 (100)
*Schulstufe: n (%)*^*a*^			
Grundstufe (Klasse 1–4)	–	652 (42,9)	–
Unterstufe (Klasse 5–6)	249 (24,3)	306 (20,1)	184 (27,4)
Mittelstufe (Klasse 7–9)	423 (41,3)	375 (24,7)	316 (47,0)
Oberstufe (Klasse 10–12)	352 (34,4)	187 (12,3)	172 (25,6)
*Fälle pro Erhebungszeitraum: n (%)*			
11/2020–12/2020	592 (57,8)	1160 (72,2)	–
02/2021–04/2021	432 (42,2)	446 (27,8)	–

*n* absolute Häufigkeiten, *%* prozentuale Häufigkeiten, *M* Mittelwert, *SD* Standardabweichung

^a^Aufgrund von Missings variiert die absolute Anzahl

### Stärke der psychischen Be- und Entlastungen

Die Ausprägung der Be- und Entlastungen durch die COVID-19-Pandemie auf Itemebene in der *Klinikstichprobe *sowohl nach Angaben der Bezugspersonen (*n* = 254, 81,5 % Mütter, 15,4 % Väter, 3,1 % andere) als auch im Patient:innenurteil ab 11 Jahren (*n* = 141) zeigt Tab. 4.

**Table 4:** 

	Urteil	*M*	*SD*	*n (%)*				
				Viel besser (−2)	Etwas besser (−1)	Unverändert (0)	Etwas schlechter (1)	Viel schlechter (2)
*01 Beziehungen Kind zu Familienmitgliedern*	E	0,00	0,87	11 (4,3)	50 (19,7)	132 (52,0)	49 (19,3)	12 (4,7)
	K	0,14	0,87	3 (2,1)	22 (15,6)	70 (49,6)	44 (31,2)	2 (1,4)
*02 Beziehungen Kind zu Freund:innen*	E	0,60	0,80	1 (0,4)	10 (3,9)	115 (45,3)	91 (35,8)	37 (14,6)
	K	0,44	0,91	5 (3,5)	11 (7,8)	57 (40,4)	53 (37,6)	15 (10,6)
*03 Belastungen durch Schule/Lernen*	E	0,42	1,08	13 (5,1)	34 (13,4)	84 (33,1)	79 (31,1)	44 (17,3)
	K	0,58	1,05	3 (2,1)	22 (15,6)	35 (24,8)	52 (36,9)	29 (20,6)
*04 Betreuung des Kindes*	E	0,40	0,90	5 (2,0)	28 (11,0)	112 (44,1)	79 (31,1)	30 (11,8)
	K	–	–	–	–	–	–	–
*05 Freizeit/Beschäftigungsmöglichkeit*	E	1,42	0,84	3 (1,2)	3 (1,2)	32 (12,6)	62 (24,4)	154 (60,6)
	K	1,16	0,93	1 (0,7)	5 (3,5)	30 (21,3)	39 (27,7)	66 (46,8)
*06 Gereiztheit/Unruhe/Unausgeglichenheit*	E	0,50	0,99	5 (2,0)	37 (14,6)	79 (31,1)	93 (36,6)	40 (15,7)
	K	0,65	0,89	4 (2,8)	8 (5,7)	41 (29,1)	69 (48,9)	19 (13,5)
*07 Ängstlichkeit/Unsicherheit/Traurigkeit*	E	0,41	0,82	4 (1,6)	15 (5,9)	134 (52,8)	74 (29,1)	27 (10,6)
	K	0,57	0,94	3 (2,1)	9 (6,4)	60 (42,6)	42 (29,8)	27 (19,1)
*08 Berufliche Situation für Bezugsperson*	E	0,25	0,76	3 (1,2)	21 (8,3)	159 (62,6)	52 (20,5)	19 (7,5)
	K	–	–	–	–	–	–	–
*09 Familiäre Situation*	E	0,17	0,75	5 (2,0)	27 (10,6)	154 (60,6)	57 (22,4)	11 (4,3)
	K	–	–	–	–	–	–	–
*10 Veränderungen für Kind insgesamt*	E	0,83	0,95	3 (1,2)	27 (10,6)	41 (16,1)	123 (48,4)	60 (23,6)
	K	0,71	0,96	3 (2,1)	14 (9,9)	31 (22,0)	66 (46,8)	27 (19,1)
*11 Veränderungen für Bezugsperson insgesamt*	E	1,02	0,85	4 (1,6)	10 (3,9)	35 (13,8)	134 (52,8)	71 (28,0)
	K	–	–	–	–	–	–	–
*CBB-Gesamtbelastungsindex*	E	0,55	0,52					
	E (C)	0,60	0,60					
	K	0,61	0,54					

*n* absolute Häufigkeiten, *%* prozentuale Häufigkeiten, *M* Mittelwert, *SD* Standardabweichung, *E* Eltern, *K* Kind, *E (C)* CBB-E-Cross-Informant-Index, enthält die im Eltern- und Selbsturteil korrespondierenden Items

Die Einschätzungen der Eltern ergeben, dass die Pandemie insgesamt bei fast 24 % der Patient:innen zu sehr starken und bei weiteren 48 % zu leichteren negativen Veränderungen geführt hat (Item 10). Insgesamt trat also bei rund 72 % eine vermehrte Belastung auf, während für rund 16 % der Patient:innen keine Veränderungen und für weitere knapp 12 % leichte oder sogar starke Verbesserungen, d. h. Entlastungen, angegeben wurden.

Insbesondere die Freizeit- und Beschäftigungsmöglichkeiten der Patient:innen (Item 5) haben sich nach Angabe der Bezugspersonen durch die Pandemie verschlechtert: Etwa 24 % der Bezugspersonen berichteten eine leichte und fast 61 % eine starke Verschlechterung der Freizeit- und Beschäftigungsmöglichkeiten ihrer Kinder. Die Beziehungen des Kindes zu anderen Familienmitgliedern (Item 1) haben sich laut der Bezugspersonen hingegen bei 52 % der Patient:innen nicht verändert, bei rund 24 % leicht oder stark verbessert und bei ebenfalls 24 % leicht oder stark verschlechtert. Für die Bezugspersonen selbst verschlechterte sich die Gesamtsituation nach eigener Einschätzung im Verlauf der Pandemie noch etwas stärker als bei ihren Kindern (ca. 81 %, Item 11).

Vergleichbar zu den Eltern gaben auch rund 19 % der Kinder und Jugendlichen an, dass es ihnen durch die Coronapandemie sehr viel schlechter, und fast 47 %, dass es ihnen ein wenig schlechter gehe (Item 10). Auch die Kinder und Jugendlichen schätzten besonders die Freizeit- und Beschäftigungsmöglichkeiten als eher negativ verändert ein.

Gereiztheit, Unruhe und Unausgeglichenheit der Kinder oder Jugendlichen (Item 6) nahmen nach dem Eltern- und Selbsturteil in rund 14–16 % der Fälle stark zu, wobei leichte Verschlechterungen noch deutlich häufiger berichtet wurden. Allerdings gibt es auch Fälle, bei denen sich diese Probleme während der Pandemie verbesserten. Ähnlich sieht es bei Ängstlichkeit, Unsicherheit und Traurigkeit (Item 7) aus, die sich bei etwa 11–19 % der Fälle nach dem Eltern- bzw. Selbsturteil stark verschlechterten. Verbesserungen traten hier laut Elternurteil aber deutlich seltener auf.

In der Substichprobe, in der die CBB-Beurteilungen sowohl von Eltern als auch von Patient:innen vorliegen (Onlinematerial 4), gibt es keinen signifikanten Unterschied im Eltern- (*M* = 0,65, *SD* = 0,56) und Selbsturteil (*M* = 0,61, *SD* = 0,54). Die beiden korrespondierenden Beurteilungen korrelieren zu *r* = 0,53 (*p* < 0,001).

Die Ausprägungen der Be- und Entlastungen im Zusammenhang mit der Coronapandemie nach Angaben der Bezugspersonen (*n* = 1606, 78,7 % Mütter, 20,7 % Väter, 0,6 % andere) und der Kinder und Jugendlichen ab 11 Jahren (*n* = 1024) in der *(Vor‑)Schulstichprobe *zeigt Tab. 5. Im Vergleich zur *Klinikstichprobe* (Tab. 4) haben sich laut der Eltern für Kinder und Jugendliche im Verlauf der Pandemie insgesamt etwas seltener, bei knapp 17 % (Vergleich Klinikstichprobe: knapp 24 %) sehr starke und bei weiteren gut 61 % leichtere negative Veränderungen ergeben. Insgesamt führte die Pandemie also bei rund 78 % (Klinikstichprobe: rund 72 %) zu vermehrter Belastung, während der Zustand bei etwa 22 % laut Elternurteil unverändert war. In den Mittelwerten unterscheiden sich beide Stichproben auch nominell kaum.

**Table 5:** 

	Urteil	*M*	*SD*	*n (%)*				
				Viel besser (−2)	Etwas besser (−1)	Unverändert (0)	Etwas schlechter (1)	Viel schlechter (2)
*01 Beziehungen Kind zu Familienmitgliedern*	E	−0,03	0,65	33 (2,1)	236 (14,7)	1109 (69,2)	200 (12,5)	25 (1,6)
	K	0,00	0,69	26 (2,5)	147 (14,4)	661 (64,8)	171 (16,8)	15 (1,5)
*02 Beziehungen Kind zu Freund:innen*	E	0,45	0,75	13 (0,8)	94 (5,9)	770 (48,0)	610 (38,1)	116 (7,2)
	K	0,36	0,91	30 (2,9)	140 (13,7)	364 (35,5)	414 (40,4)	76 (7,4)
*03 Belastungen durch Schule/Lernen*	E	0,56	0,86	18 (1,1)	138 (8,7)	576 (36,2)	653 (41,1)	204 (12,8)
	K	0,48	0,96	29 (2,8)	125 (12,2)	327 (32,0)	415 (40,6)	127 (12,4)
*04 Betreuung des Kindes*	E	0,44	0,81	23 (1,4)	79 (5,0)	832 (52,2)	498 (30,7)	117 (10,7)
	K	–	–	–	–	–	–	–
*05 Freizeit/Beschäftigungsmöglichkeit*	E	1,50	0,72	9 (0,6)	15 (0,9)	118 (7,4)	490 (30,5)	973 (60,6)
	K	1,29	0,90	16 (1,6)	37 (3,6)	103 (10,1)	345 (33,7)	523 (51,1)
*06 Gereiztheit/Unruhe/Unausgeglichenheit*	E	0,49	0,75	11 (0,7)	93 (5,8)	720 (45,0)	657 (41,1)	118 (7,4)
	K	0,46	0,78	12 (1,2)	77 (7,5)	430 (42,1)	432 (42,3)	71 (6,9)
*07 Ängstlichkeit/Unsicherheit/Traurigkeit*	E	0,35	0,65	14 (0,9)	55 (3,4)	950 (59,2)	523 (32,6)	62 (3,9)
	K	0,32	0,75	18 (1,8)	61 (6,0)	575 (56,2)	310 (30,3)	59 (5,8)
*08 Berufliche Situation für Bezugsperson*	E	0,42	0,81	29 (1,8)	76 (4,8)	865 (54,1)	460 (28,8)	170 (10,6)
	K	–	–	–	–	–	–	–
*09 Familiäre Situation*	E	0,14	0,73	34 (2,1)	174 (10,9)	993 (61,9)	343 (21,4)	59 (3,7)
	K	–	–	–	–	–	–	–
*10 Veränderungen für Kind insgesamt*	E	0,91	0,72	4 (0,2)	62 (3,9)	283 (17,6)	984 (61,3)	271 (16,9)
	K	0,55	0,85	20 (2,0)	104 (10,2)	269 (26,4)	546 (53,5)	81 (7,9)
*11 Veränderungen für Bezugsperson insgesamt*	E	1,04	0,78	20 (1,2)	51 (3,2)	192 (12,0)	931 (58,0)	411 (25,6)
	K	–	–	–	–	–	–	–
*CBB-Gesamtbelastungsindex*	E	0,57	0,44					
	E (C)	0,60	0,46					
	K	0,50	0,48					

*n* absolute Häufigkeiten, *%* prozentuale Häufigkeiten, *M* Mittelwert, *SD* Standardabweichung, *E* Eltern, *K* Kind,* E (C)* CBB-E-Cross-Informant-Index

Ähnlich wie in der *Klinikstichprobe* haben sich insbesondere die Freizeit- und Beschäftigungsmöglichkeiten der Kinder nach Angabe der Bezugspersonen durch die COVID-19-Pandemie verschlechtert. Die Beziehungen der Kinder und Jugendlichen zu anderen Familienmitgliedern waren hingegen nach Einschätzung der Bezugsperson bei rund 69 % unverändert, bei rund 17 % verbessert und bei ca. 14 % verschlechtert. Im Verlaufe der Pandemie verschlechterte sich die Gesamtsituation für die Bezugspersonen selbst noch etwas stärker als für deren Kinder (84 %).

Im Vergleich zu den Eltern gaben nur rund 8 % der Kinder und Jugendlichen an, dass es ihnen durch die Coronapandemie sehr viel schlechter, und fast 54 %, dass es ihnen ein wenig schlechter gehe. Auch die Kinder und Jugendlichen schätzten besonders die Freizeit- und Beschäftigungsmöglichkeiten durch die Pandemie als negativ verändert ein. Im Unterschied zur *Klinikstichprobe* beschrieben in der *(Vor‑)Schulstichprobe* rund 45 % der Eltern keine Veränderungen hinsichtlich gereizten, unruhigen oder unausgeglichenen Verhaltens bei ihren Kindern. Im Selbsturteil zeigt sich ein ähnliches Ergebnis. Ebenfalls geringer als in der *Klinikstichprobe* wurde eine Zunahme von Ängstlichkeit, Unsicherheit und Traurigkeit empfunden, hier gaben nur knapp 36 % der Schüler:innen negative Veränderungen an.

In der Substichprobe der *(Vor‑)Schulstichprobe *(*n* = 672), in der die CBB-Beurteilungen im Eltern- und im Selbsturteil vorliegen (Onlinematerial 5), unterscheiden sich die Beurteilungen (CBB-Cross-Informant-Index) von Eltern (*M* = 0,58, *SD* = 0,46) und Schüler:innen (*M* = 0,47, *SD* = 0,47) signifikant (*t*(671) = 5,928, *p* < 0,001). Die beiden korrespondierenden Beurteilungen korrelieren zu *r* = 0,47 (*p* < 0,001).

Mit einer Effektstärke von Cohens *d* = 0,5 weisen Patient:innen der *Klinikstichprobe* (*M* = 0,61, *SD* = 0,54) nach eigenen Angaben eine etwas höhere Belastung auf als die Kinder der *(Vor‑)Schulstichprobe* (*M* = 0,50, *SD* = 0,48). Auf der Ebene der Elternurteile lässt sich hingegen so gut wie kein Unterschied zwischen den beiden Stichproben nachweisen (Cohens *d* = 0,1).

### Psychische Belastung und soziodemografische Parameter sowie Veränderungen im Umfeld

Zwischen soziodemografischen Merkmalen der Familien und der Gesamtbelastung durch die Pandemie zeigt sich in der *Klinikstichprobe* kein signifikanter Zusammenhang: Weder zum Alter der Patient:innen noch zu ihrem Geschlecht, zum sozioökonomischen Status der Familie, zur Familienzusammensetzung („getrennt“ versus „zusammenlebend“) oder zum Vorhandensein psychischer oder körperlicher Erkrankungen bei weiteren Familienmitgliedern konnte ein statistisch signifikanter Zusammenhang mit dem Gesamtbelastungsindex im Eltern- oder im Selbsturteil gefunden werden. Auch in der *(Vor‑)Schulstichprobe* zeigen sich kaum bedeutsame Zusammenhänge zwischen soziodemografischen Merkmalen und der Gesamtbelastung durch die COVID-19-Pandemie. Lediglich zwischen Alter und dem Gesamtbelastungsindex im Selbsturteil kann ein schwacher, aber signifikanter Zusammenhang (*r* = 0,13, *p* < 0,01) gefunden werden. Dahingegen korrelieren die subjektiv erlebten Veränderungen der familiären Rahmenbedingungen (CBB-E-Rahmenbedingungen) in beiden Stichproben statistisch signifikant mit den angegebenen Belastungen im Eltern- (CBB-Cross-Informant-Index) und Selbsturteil. Es konnte dementsprechend eine Assoziation zwischen ungünstigen Veränderungen familiärer Rahmenbedingungen durch die Pandemie und stärkeren von den Eltern (Klinikstichprobe: *r* = 0,54, *p* < 0,001; (Vor‑)Schulstichprobe: *r* = 0,57, *p* < 0,001) und Kindern bzw. Jugendlichen beurteilten Belastungen (Klinikstichprobe: *r* = 0,26, *p* < 0,005; (Vor‑)Schulstichprobe: *r* = 0,20, *p* < 0,001) gefunden werden.

### Psychische Belastung und klinische Diagnosen sowie emotionale und Verhaltensauffälligkeiten

In der *Klinikstichprobe* unterscheiden sich die einzelnen Diagnosegruppen (internale Störungen, externale Störungen, gemischte Störungen, andere Störungen) hinsichtlich der Belastung während der Pandemie weder im Eltern- (*F*(3, 250) = 1,61, *p* = 0,19) noch im Selbsturteil (*F*(3, 137) = 1,37, *p* = 0,26). Allerdings lassen sich geringe, aber statistisch signifikante Zusammenhänge zwischen der von den Eltern bzw. den Kindern und Jugendlichen beurteilten psychischen Belastung und emotionalen und Verhaltensauffälligkeiten insgesamt nachweisen (CBCL-Gesamtauffälligkeit: *r* = 0,20, *p* < 0,05; YSR-Gesamtauffälligkeit: *r* = 0,25, *p* < 0,01). Die Zusammenhänge lassen sich ebenso für viele Subskalen des CBCL bzw. YSR belegen (s. Onlinematerial 6). Auch zwischen Belastung und Funktionsbeeinträchtigungen, einschließlich subjektiven Leidensdrucks, im Eltern- bzw. Selbsturteil lassen sich signifikante Zusammenhänge finden (FBB-SCREEN:* r* = 0,13, *p* < 0,05; SBB-SCREEN: *r* = 0,36, *p* < 0,01).

Der Anteil der Kinder und Jugendlichen mit emotionalen und Verhaltensauffälligkeiten in der *(Vor‑)Schulstichprobe* liegt bei Anwendung der vorgegeben Cut-offs mit 11,4 % (Elternurteil) bzw. 10,0 % (Selbsturteil) im Bereich der durch die Normierungen dieser Instrumente erwarteten Auffälligkeitsraten [26, 27]. Auch in der *(Vor‑)Schulstichprobe* lassen sich ähnlich hohe Zusammenhänge zwischen der von den Eltern bzw. den Kindern und Jugendlichen beurteilten psychischen Belastung und emotionalen und Verhaltensauffälligkeiten insgesamt nachweisen (SDQ‑E Gesamtauffälligkeit: *r* = 0,33, *p* < 0,01; SDQ‑S Gesamtauffälligkeit: *r* = 0,22, *p* < 0,01). Die Zusammenhänge lassen sich auch für fast alle Subskalen des SDQ‑E bzw. SDQ‑S belegen.

## Diskussion

Die vorliegende multizentrische Analyse untersucht das Ausmaß der psychischen Belastung von Kindern und Jugendlichen und ihren Familien während der COVID-19-Pandemie und den Zusammenhang mit emotionalen und Verhaltensauffälligkeiten, mit negativ erlebten Veränderungen im Umfeld sowie mit soziodemografischen Parametern. Dazu wurden die Daten aus 2 Stichproben parallel analysiert: eine klinische Inanspruchnahmestichprobe aus kinder- und jugendpsychiatrischen sowie kinder- und jugendlichenpsychotherapeutischen Ambulanzen und eine Feldstichprobe von Kindern und Jugendlichen, die vorschulische und schulische Einrichtungen in verschiedenen Städten Deutschlands besuchen. Zur Berücksichtigung verschiedener Beurteilungsperspektiven wurden sowohl Eltern- als auch Selbsturteile von Kindern und Jugendlichen ab 11 Jahren erfasst.

Insgesamt lagen die Belastungsindizes auf einer Skala von −2 (starke Entlastung) bis +2 (starke Belastung) in beiden Stichproben und unter beiden Beurteilungsperspektiven im Mittel zwischen 0,5 und 0,6. Die Unterschiede zwischen den Beurteilenden und auch zwischen beiden Stichproben sind weitgehend zu vernachlässigen, d. h., sowohl bei einer überwiegend unauffälligen *(Vor‑)Schulstichprobe* als auch bei einer psychisch auffälligen *Klinikstichprobe* lassen sich im Durchschnitt leichte bis moderate Belastungen im Verlauf der Pandemie aus den Berichten von Eltern und ihren Kindern erkennen. Lediglich im Urteil der Kinder und Jugendlichen konnte in der *Klinikstichprobe* eine signifikante und gering bis moderat erhöhte Belastung im Vergleich zur *(Vor‑)Schulstichprobe* festgestellt werden. Aus diesem Befund lässt sich jedoch nicht schlussfolgern, dass bei allen Kindern und Familien nur leichte Belastungen zu eruieren sind, wie eine detaillierte Analyse der Daten zeigt. Insgesamt beschreiben rund 60–70 % der Eltern und der Kinder und Jugendlichen sowohl in der klinischen als auch in der Feldstichprobe eine Zunahme an Belastungen, wobei eine starke Belastung bei rund 10–25 % der Fälle festzustellen ist. Dieses Ergebnis korrespondiert mit dem Ergebnis der bundesweit repräsentativen Studie von Ravens-Sieberer et al. [12, 13], nach der 71 % der Kinder und Jugendlichen und 75 % der Eltern eine Belastung durch die erste Pandemiewelle angaben. In der vorliegenden Studie kann das Ausmaß der Belastung dahin gehend differenziert werden, dass in der Mehrzahl der Fälle eine geringe bis moderate Belastung berichtet wird.

Darüber hinaus beschreiben rund 12 % der Eltern und der Kinder und Jugendlichen in der *Klinikstichprobe* eine Entlastung für das Kind/den Jugendlichen während der Pandemie. In der *(Vor‑)Schulstichprobe* empfinden das auch rund 12 % der Kinder und Jugendlichen so, aber nur 4 % der Eltern. Rund 17–24 % der Eltern oder Kinder und Jugendlichen in beiden Stichproben berichten von einer Verbesserung der familiären Beziehungen während der Pandemie. Diese Ergebnisse entsprechen den Ergebnissen von Cost et al. [20], nach denen bei 19–31 % der Kinder und Jugendlichen in mindestens einem Bereich eine Verbesserung der psychischen Gesundheit beschrieben wird. Eine Entlastung durch geringere schulische Anforderungen aufgrund der Beschulung im häuslichen Umfeld dürfte allerdings nur passager sein.

Auch hinsichtlich einzelner Belastungsfaktoren lassen sich vergleichbare Ergebnisse bei der *(Vor‑)Schulstichprobe* und der COPSY-Studie belegen: So beschreiben rund 53 % der Kinder und Jugendlichen höhere Belastungen bezüglich Schule und Lernen (COPSY: 64 %) und 48 % beschreiben eine Verschlechterung der Beziehungen zu ihren Freund:innen (COPSY: 39 %). Diese Ähnlichkeiten sind ein weiterer Hinweis auf die Repräsentativität der Ergebnisse aus der *(Vor‑)Schulstichprobe*. Die mit Abstand stärksten Belastungen sehen sowohl Eltern als auch Kinder und Jugendliche durch die reduzierten Freizeit- und Beschäftigungsmöglichkeiten – hier beschreiben rund 70–90 % leichte bis starke Verschlechterungen.

Die psychischen Auffälligkeiten in Form von Gereiztheit, Unruhe und Unausgeglichenheit oder in Form von Ängstlichkeit, Unsicherheit oder Traurigkeit haben sich in der *Klinikstichprobe* sowohl im Eltern- als auch im Selbsturteil im Vergleich zur Feldstichprobe etwa doppelt so häufig stark verschlechtert. Dies weist hin auf eine erhöhte Vulnerabilität für eine stärkere Zunahme von emotionalen und Verhaltensauffälligkeiten in der *Klinikstichprobe* bei im Durchschnitt vergleichbar starker, d. h. überwiegend leichter bis moderater Belastungszunahme durch die Pandemie. Dies entspricht auch der Einschätzung der Direktor:innen kinder- und jugendpsychiatrischer Kliniken in Europa [31], die ebenfalls eine moderate Belastung der Patient:innen und eine Zunahme der Inanspruchnahme berichten.

Zwar konnten im Gegensatz zu manchen anderen Studien (z. B. [12, 13]) keine substanziellen soziodemografischen Faktoren gefunden werden (z. B. Alter, Geschlecht, sozioökonomischer Status), welche die Belastung durch COVID-19 deutlich beeinflussen; allerdings zeigen sich leichte bis moderate Zusammenhänge zwischen der subjektiv erlebten Verschlechterung der familiären und sozialen Situation und einem erhöhten Belastungserleben.

Die COPSY-Studie [13] berichtet von erhöhten psychischen Auffälligkeiten in der ersten Pandemiephase im Elternurteil (17,8 %) im Vergleich zu einer Erhebung vor Ausbruch von COVID-19-Pandemie. Unter Verwendung des gleichen Fragebogenverfahrens (SDQ-Elternurteil) konnten in der *(Vor‑)Schulstichprobe* mit 11,4 % allerdings keine so deutlich erhöhten Werte festgestellt werden. Dies mag mit dem Erhebungszeitpunkt (Pandemiewelle 2 bis Mitte Pandemiewelle 3) oder auch mit einer begrenzten Repräsentativität der vorliegenden *(Vor‑)Schulstichprobe* zusammenhängen.

Schließlich konnten auch leichte bis moderate Zusammenhänge zwischen Belastungen und psychischen Auffälligkeiten sowohl im Selbst- als auch im Fremdurteil in beiden Stichproben nachgewiesen werden, wobei die Beeinflussungsrichtungen aus diesen Korrelationen nicht erschlossen werden können: Belastungen können zu stärkeren psychischen Auffälligkeiten beitragen und/oder stärkere psychische Auffälligkeiten können Belastungen erhöhen. Grundsätzlich handelt es sich um Querschnittserhebungen, somit lassen die vorgenommenen Korrelationsanalysen keine Ableitung kausaler Zusammenhänge zu.

Neben den Stärken der Studie – vor allem die parallele Betrachtung von Eltern- und Selbsteinschätzungen in einer Feldstichprobe und einer Klinikstichprobe – sind einige Limitationen zu beachten. Trotz Bemühungen, Daten konsekutiv und multizentrisch zu erheben, um eine möglichst hohe Repräsentativitätsrate in der klinischen Inanspruchnahmestichprobe zu erreichen, gelang dies aufgrund von hohen „Schwundraten“ und stark unterschiedlichen Teilnahmequoten der einzelnen Standorte nicht. Da eine weitere Fragestellung der Studie sich auf die Durchführung von Ferntherapie bezog, wurden außerdem Patient:innen mit einer Kontraindikation für Videotherapie nicht in die Klinikstichprobe aufgenommen. Dadurch wurden Patient:innen mit stark ausgeprägten psychischen Störungen nicht berücksichtigt. Insofern ist die Repräsentativität der *Klinikstichprobe* eingeschränkt. Auch die Repräsentativität der Feldstichprobe ist vor allem durch die lokale Verteilung und die unterschiedlich hohen Teilnahmequoten in den Einrichtungen eingeschränkt. Die Studie ist lediglich eine Momentaufnahme während des zweiten Lockdowns, die Aussagen bezüglich subjektiv erlebter Belastungen ermöglicht. Das Ausmaß der objektiven Einschränkungen wurde nicht erhoben und Aussagen über die Stabilität der identifizierten Belastungen können nicht getroffen werden. Außerdem wurden beide Stichproben nicht über statistische Verfahren direkt miteinander verglichen und Unterschiede in der Zusammensetzung beider Stichproben wurden nicht korrigiert. Mit dieser Publikation sollten die basalen Ergebnisse für jede Stichprobe dargestellt werden. Komplexere Analysen sind weiteren Publikationen vorbehalten.

## Fazit

Die Ergebnisse der Studie unterstreichen die Notwendigkeit während einer Pandemie, belastete Subgruppen sowohl in klinischen Stichproben als auch in der Gesamtbevölkerung zu identifizieren und gezielte Interventionen anzubieten. Wegen des hohen Anteils von Kindern und Jugendlichen ohne ausgeprägte Belastung sind universelle Interventionen nicht indiziert.

## Supplementary Information





## Footnotes

1 https://www.netzwerk-universitaetsmedizin.de/.
