# Supplementary material for: The mental burden of children, adolescents, and their families during the COVID-19 pandemic and associations with emotional and behavioral problems
Source: Bundesgesundheitsblatt Gesundheitsforschung Gesundheitsschutz. 2021 Nov 9;64(12):1522–32. [Article in German] doi: 10.1007/s00103-021-03455-1 (PMC8576319; doi:10.1007/s00103-021-03455-1)
Supplement: Supplementary file 1 [file 103_2021_3455_MOESM1_ESM.pdf]

Bundesgesundheitsblatt 12/2021

Onlinematerial zum Beitrag:

Die psychische Belastung von Kindern, Jugendlichen und ihren Familien während der COVID-19-Pandemie und der Zusammenhang mit emotionalen und Verhaltensauffälligkeiten

Manfred Döpfner[1,2], Julia Adam[1], Carolina Habbel[1], Birte Schulte[2], Karen Schulze-Husmann[1], Michael Simons[3], Fabiola Heuer[4], Christiane Wegner[5], Stephan Bender[2], TEMPO-Studiengruppe, B-FAST-Studiengruppe

[1] Ausbildungsinstitut für Kinder- und Jugendlichenpsychotherapie (AKiP), Medizinische Fakultät und Uniklinik Köln, Universität zu Köln, Köln, Deutschland
[2] Klinik für Psychiatrie, Psychosomatik und Psychotherapie des Kindes- und Jugendalters, Medizinische Fakultät und Uniklinik Köln, Universität zu Köln, Köln, Deutschland
[3] Klinik für Psychiatrie, Psychosomatik und Psychotherapie des Kindes- und Jugendalters, RWTH Aachen, Aachen, Deutschland
[4] Klinik für Psychiatrie, Psychosomatik und Psychotherapie des Kindes- und Jugendalters, Charité, Universitätsmedizin Berlin, Berlin, Deutschland
[5] Klinik für Kinder- und Jugendpsychiatrie/Psychotherapie, Universitätsmedizin Göttingen, Georg-August-Universität Göttingen, Göttingen, Deutschland

Korrespondenzadresse

Prof. Dr. Manfred Döpfner, Dipl.-Psych.
Klinik und Poliklinik für Psychiatrie und Psychotherapie des Kindes- und Jugendalters Universitätsklinik Köln
Robert-Koch-Str. 10
50931 Köln
Deutschland
manfred.doepfner@uk-koeln.de

Inhalte

Onlinematerial 1: Corona-Belastungsbögen
Onlinematerial 2: Weitere Informationen zu den Messinstrumenten
Onlinematerial 3: Ergebnisse von vergleichenden Analysen innerhalb der Klinikstichprobe und der (Vor-)Schulstichprobe
Onlinematerial 4: Stärke der Belastungen durch die Corona-Pandemie in der Klinikstichprobe (Eltern- und Selbsturteil vorliegend; n = 141), Angaben auf Item-Ebene
Onlinematerial 5: Stärke der Belastungen durch die Corona-Pandemie in der (Vor-)Schulstichprobe
Onlinematerial 6: Signifikante Korrelationen mit korrespondierendem CBB-Gesamtindizes b6

Onlinematerial 1: Corona-Belastungsbögen

M. Döpfner, A. Görtz-Dorten (2020), Corona-Belastungsbogen. Klinik für Psychiatrie, Psychosomatik und Psychotherapie des Kindes- und Jugendalters an der Uniklinik Köln. Modifizierte Fassung des Bogens von T. Jans, Übernahme von Items aus dem CRISIS-Bogen, Stand 2020.05.18

Corona Belastungsbogen – Eltern (CBB-E)

A) Aktuelle Veränderungen von Beziehungen und Belastungen im Zusammenhang mit der Corona-Krise

1. Haben sich die Beziehungen zwischen Ihrem Kind und den anderen Familienmitgliedern im Zusammenhang mit der Corona-Krise verändert?
   a. ☐ Viel schlechter
   b. ☐ Ein wenig schlechter
   c. ☐ Gleichgeblieben
   d. ☐ Etwas besser
   e. ☐ Viel besser

2. Haben sich die Beziehungen Ihres Kindes zu seinen Freundinnen oder Freunden im Zusammenhang mit der Corona-Krise verändert?
   a. ☐ Viel schlechter
   b. ☐ Ein wenig schlechter
   c. ☐ Gleichgeblieben
   d. ☐ Etwas besser
   e. ☐ Viel besser

3. Haben sich die Belastungen für Ihr Kind durch Schule und Lernen im Zusammenhang mit der Corona-Krise verändert?
   a. ☐ Viel schlechter
   b. ☐ Ein wenig schlechter
   c. ☐ Gleichgeblieben
   d. ☐ Etwas besser
   e. ☐ Viel besser

4. Hat sich die Betreuung bzw. Beaufsichtigung tagsüber für Ihr Kind im Zusammenhang mit der Corona-Krise verändert?
   a. ☐ Viel schlechter
   b. ☐ Ein wenig schlechter
   c. ☐ Gleichgeblieben
   d. ☐ Etwas besser
   e. ☐ Viel besser

5. Haben sich die Freizeit- oder Beschäftigungsmöglichkeiten für Ihr Kind im Zusammenhang mit der Corona-Krise verändert?
   a. ☐ Viel schlechter
   b. ☐ Ein wenig schlechter
   c. ☐ Gleichgeblieben
   d. ☐ Etwas besser
   e. ☐ Viel besser

B) Aktuelle Veränderungen des Verhaltens der/s Kindes/Jugendlichen im Zusammenhang mit der Corona-Krise

6. Haben Sie den Eindruck, dass Ihr Kind während der Corona-Krise gereizter, unruhiger oder unausgeglichener geworden ist?
   a. ☐ Ja, mein Kind ist viel gereizter, unruhiger oder unausgeglichener
   b. ☐ Ja, mein Kind etwas gereizter, unruhiger oder unausgeglichener
   c. ☐ Bei meinem Kind hat sich nichts verändert
   d. ☐ Mein Kind ist weniger gereizt, unruhig oder unausgeglichen
   e. ☐ Mein Kind ist viel weniger gereizt, unruhig oder unausgeglichen

7. Haben Sie den Eindruck, dass Ihr Kind während der Corona-Krise ängstlicher, unsicherer oder trauriger geworden ist?
   a. ☐ Ja, mein Kind ist viel ängstlicher, unsicherer oder trauriger
   b. ☐ Ja, mein Kind ist etwas ängstlicher, unsicherer oder trauriger
   c. ☐ Bei meinem Kind hat sich nichts verändert
   d. ☐ Mein Kind ist weniger ängstlich, unsicher oder traurig
   e. ☐ Mein Kind ist viel weniger ängstlich, unsicher oder traurig

C) Aktuelle Veränderungen der familiären und der beruflichen Situation im Zusammenhang mit der Corona-Krise

8. Hat sich die berufliche Situation für Sie im Zusammenhang mit der Corona-Krise verändert?
   a. ☐ Viel schlechter
   b. ☐ Ein wenig schlechter
   c. ☐ Gleichgeblieben
   d. ☐ Etwas besser
   e. ☐ Viel besser

9. Hat sich die familiäre Situation im Zusammenhang mit der Corona-Krise verändert?
   a. ☐ Viel schlechter
   b. ☐ Ein wenig schlechter
   c. ☐ Gleichgeblieben
   d. ☐ Etwas besser
   e. ☐ Viel besser

D) Beurteilung der Veränderungen insgesamt

10. Wie fanden Sie die Veränderungen für Ihr Kind durch die Corona-Krise insgesamt?
    a. ☐ Sehr belastend
    b. ☐ Etwas belastend
    c. ☐ Weder belastend noch entlastend
    d. ☐ Etwas entlastend
    e. ☐ Sehr entlastend

11. Wie fanden Sie die Veränderungen für sich selbst durch die Corona-Krise insgesamt?
    a. ☐ Sehr belastend
    b. ☐ Etwas belastend
    c. ☐ Weder belastend noch entlastend
    d. ☐ Etwas entlastend
    e. ☐ Sehr entlastend

Corona Belastungsbogen – Kinder und Jugendliche (CBB-KJ)

1. Haben sich die Beziehungen zwischen dir und den anderen in deiner Familie durch die Corona-Krise verändert? (z.B. wie du und deine Mutter miteinander umgeht, wie du mit deinem Vater zurechtkommst, wie du dich mit deinen Geschwistern verstehst…Ist das anders geworden?)
   - ☐ Viel schlechter
   - ☐ Ein wenig schlechter
   - ☐ Gleichgeblieben
   - ☐ Etwas besser
   - ☐ Viel besser

2. Haben sich die Beziehungen zu deinen Freundinnen oder Freunden durch die Corona-Krise verändert? (z.B. wie häufig du mit deinen Freundinnen oder Freunden Kontakt hast oder wie ihr miteinander zurechtkommt …Ist das anders geworden?)
   - ☐ Viel schlechter
   - ☐ Ein wenig schlechter
   - ☐ Gleichgeblieben
   - ☐ Etwas besser
   - ☐ Viel besser

3. Haben sich für dich die Belastungen durch Schule und Lernen durch die Corona-Krise verändert? (z.B. wie du seitdem für die Schule lernst oder wie viel du für die Schule machst…Ist das anders geworden?)
   - ☐ Viel schlechter
   - ☐ Ein wenig schlechter
   - ☐ Gleichgeblieben
   - ☐ Etwas besser
   - ☐ Viel besser

4. Haben sich die die Freizeit- oder Beschäftigungsmöglichkeiten für dich durch die Corona-Krise verändert? (z.B. wie viel du unternehmen kannst, deinen Hobbies oder Lieblingsbeschäftigungen nachgehen kannst, dich verabreden und mit anderen treffen kannst…Ist das anders geworden?)?
   - ☐ Viel schlechter
   - ☐ Ein wenig schlechter
   - ☐ Gleichgeblieben
   - ☐ Etwas besser
   - ☐ Viel besser

5. Hast Du den Eindruck, dass du während der Corona-Krise gereizter, unruhiger oder unausgeglichener geworden bist?
   - ☐ Ja, ich bin viel gereizter, unruhiger oder unausgeglichener
   - ☐ Ja, ich bin etwas gereizter, unruhiger oder unausgeglichener
   - ☐ Bei mir hat sich nichts verändert
   - ☐ Ich bin etwas weniger gereizt, unruhig oder unausgeglichen
   - ☐ Ich bin viel weniger gereizt, unruhig oder unausgeglichen

6. Hast Du den Eindruck, dass du während der Corona-Krise ängstlicher, unsicherer oder trauriger geworden bist?
   - ☐ Ja, ich bin viel ängstlicher, unsicherer oder trauriger
   - ☐ Ja, ich bin etwas ängstlicher, unsicherer oder trauriger
   - ☐ Bei mir hat sich nichts verändert
   - ☐ Ich bin etwas weniger ängstlich, unsicher oder traurig
   - ☐ Ich bin viel weniger ängstlich, unsicher oder traurig

7. Wie ist es dir in der Corona-Krise insgesamt gegangen?
   - ☐ Viel schlechter
   - ☐ Ein wenig schlechter
   - ☐ Gleichgeblieben
   - ☐ Etwas besser
   - ☐ Viel besser

Onlinematerial 2: Weitere Informationen zu den Messinstrumenten

Child Behavior Checklist (CBCL) & Youth Self Report (YSR).

Beide Fragebogen erfassen in 113 (CBCL) bzw. 112 (YSR) Items auf einer dreistufigen Skala (0 = “nicht zutreffend” bis 2 = “genau oder häufig zutreffend”) emotionale und Verhaltensprobleme, die sich jeweils zu acht Problemskalen zusammenfassen lassen und aus denen drei übergeordnete Skalen (Gesamtauffälligkeit, Internale Probleme, Externale Probleme) gebildet werden. Reliabilität und faktorielle Validität der Fragebogen konnten in vielen Studien bestätigt werden [1].

Strengths and Difficulties Questionnaire (SDQ).

Der SDQ umfasst 25 Items. Jedes Item wird auf einer dreistufigen Skala (0 = „nicht zutreffend” bis 2 = „eindeutig zutreffend”) erfasst. Die Überprüfung der psychometrischen Eigenschaften weist für die Gesamtskalen auf eine akzeptable bis gute interne Konsistenz haben hin (SDQ-E: α = .82 [2]; SDQ-S: α = .77 [3]).

Literaturangaben

1. Döpfner M, Plück J, Kinnen C, für die Arbeitsgruppe Deutsche Child Behavior Checklist (2014) Deutsche Schulalter-Formen der Child Behavior Checklist von Thomas M. Achenbach. Elternfragebogen über das Verhalten von Kindern und Jugendlichen, (CBCL/ 6-18R), Lehrerfragebogen über das Verhalten von Kindern und Jugendlichen (TRF/6-18R), Fragebogen für Jugendliche (YSR/11-18R). Hogrefe, Göttingen
2. Woerner W, Becker A, Friedrich C, Rothenberger A, Klasen H, Goodman R, (2002) Normierung und Evaluation der deutschen Elternversion des Strengths and Difficulties Questionnaire (SDQ): Ergebnisse einer repräsentativen Felderhebung Zeitschrift für Kinder- und Jugendpsychiatrie und Psychotherapie 30:105-112. doi.org/10.1024//1422-4917.30.2.105
3. Lohbeck A, Schultheiß J, Petermann F, Petermann U (2015) Die deutsche Selbstbeurteilungsversion des Strengths and DifficultiesQuestionnaire (SDQ-Deu-S) Psychometrische Eigenschaften, Faktorenstruktur und Grenzwerte. Diagnostica 61:222-235. doi.org/10.1026/0012-1924/a000153

Onlinematerial 3: Ergebnisse von vergleichenden Analysen innerhalb der Klinikstichprobe und der (Vor-)Schulstichprobe

1. **Ergebnisse von vergleichenden Analysen zwischen den Standorten der Klinikstichprobe**

Zwischen den Standort(gruppierung-)en ergeben sich hinsichtlich der Variablen Alter (F(3, 276) = 5,68, p < .001), psychosoziales Funktionsniveau (F(3, 276) = 6,48, p < .001), somatische Erkrankung innerhalb der Familie (F(3,204) = 4,43, p = .005) sowie der Anzahl an Diagnosen (F(3,276) = 4,63, p = .004) signifikante Unterschiede.
In Bezug auf das Alter unterscheiden sich die Patient:innen aus Berlin (M = 10,72, SD = 3,66), AKiP Köln (M = 11,08, SD = 3,69) sowie Aachen/Göttingen (M = 13,18, SD = 3,68) signifikant voneinander. Das psychosoziale Funktionsniveau ist bei den Studienteilnehmenden aus Berlin (M = 4,73, SD = 1,64) bedeutsam niedriger ausgeprägt als bei denen der KJP Köln (M = 5,66, SD = 1,11). Bei den Patient:innen des AKiP Köln (M = 0,58, SD = 0,50) liegen signifikant häufiger somatische Erkrankungen innerhalb der Familie vor als bei denen der KJP Köln (M = 0,29, SD = 0,46). Weiterhin gibt es bedeutsame Unterschiede hinsichtlich der Anzahl der gestellten Diagnosen zwischen dem AKiP Köln (M = 1,44, SD = 0,59), den Unikliniken Aachen und Göttingen (M = 1,79, SD = 0,81) sowie der Charité Berlin (M = 1,82, SD = 0,73).

Für das Elternurteil liegt der CBB-Gesamtbelastungsindex in der kinder- und jugendpsychotherapeutischen Ambulanz (n = 91, M = 0,67, SD = 0,48) statistisch signifikant über dem Index in den kinder- und jugendpsychiatrischen Ambulanzen (n = 163, M = 0,48, SD = 0,53, t(252) = 2,977, p < .005 [Cohens d = 0,39]). Auch für das Selbsturteil der Kinder und Jugendlichen liegt der CBB-Gesamtbelastungsindex in der kinder- und jugendpsychotherapeutischen Ambulanz (n = 38, M = 0,77, SD = 0,43) statistisch signifikant über dem Index in den kinder- und jugendpsychiatrischen Ambulanzen (n = 103, M = 0,55, SD = 0,56, t(84,77) = 2,493, p < .05 [Cohens d = 0,42]). Innerhalb der kinder- und jugendpsychiatrischen Ambulanzen ergeben sich sowohl im Eltern- als auch im Selbsturteil für den CBB-Gesamtbelastungsindex keine statistisch bedeutsamen Unterschiede zwischen Erstvorstellungen (n = 50) und Wiedervorstellungen (n = 129).

2. **Ergebnisse von vergleichenden Analysen zwischen Substichproben der (Vor-)Schulstichprobe**

Die Kinder aus den Grundschulen (n = 652, M = 0,59, SD = 0,43) und die Kinder und Jugendlichen aus den weiterführenden Schulen (n = 937, M = 0,55, SD = 0,44) unterscheiden sich im CBB-Gesamtbelastungsindex des Elternurteils nicht statistisch signifikant. Im Elternurteil ist der CBB-Gesamtbelastungsindex im Erhebungszeitraum November/Dezember 2020 (n = 1160, M = 0,54, SD = 0,40) signifikant niedriger ausgeprägt als im Zeitraum Februar bis April 2021 (n = 446, M = 0,65, SD = 0,50, t(677,665) = -4.072, p < .001, Cohens d = 0,25). Im Selbsturteil wird die Gesamtbelastung ebenfalls im Zeitraum November/Dezember 2020 (n = 592, M = 0,45, SD = 0,46) als signifikant geringer beurteilt als im Zeitraum Februar bis April 2021 (n = 432, M = 0,55, SD = 0,50, t(1022) = -3.258, p <= .001, Cohens d = 0,21).

Onlinematerial 4: Stärke der Belastungen durch die Corona-Pandemie in der *Klinikstichprobe* (Eltern- und Selbsturteil vorliegend; *n* = 141), Angaben auf Item-Ebene

	**Urteil**	***M***	***SD***	***n (%)***				
				**viel besser (-2)**	**etwas besser (-1)**	**unverändert (0)**	**etwas schlechter (1)**	**viel schlechter (2)**
**01 Beziehungen Kind zu Familienmitgliedern**	E	0,03	0,82	3 (2,1)	29 (20,6)	77 (54,6)	25 (17,7)	7 (5,0)
	K	0,14	0,77	3 (2,1)	22 (15,6)	70 (49,6)	44 (31,2)	2 (1,4)
**02 Beziehungen Kind zu Freund:innen**	E	0,65	0,76	1 (0,7)	2 (1,4)	62 (44,0)	57 (40,4)	19 (13,5)
	K	0,44	0,91	5 (3,5)	11 (7,8)	57 (40,4)	53 (37,6)	15 (10,6)
**03 Belastungen durch Schule / Lernen**	E	0,52	1,05	5 (3,5)	19 (13,5)	40 (28,4)	52 (36,9)	25 (17,7)
	K	0,58	1,05	3 (2,1)	22 (15,6)	35 (24,8)	52 (36,9)	29 (20,6)
**04 Freizeit / Beschäftigungsmöglichkeit**	E	1,50	0,76	0 (0,0)	2 (1,4)	17 (12,1)	30 (21,3)	92 (65,2)
	K	1,16	0,93	1 (0,7)	5 (3,5)	30 (21,3)	39 (27,7)	66 (46,8)
**05 Gereiztheit / Unruhe / Unausgeglichenheit**	E	0,52	0,97	3 (2,1)	19 (13,5)	40 (28,4)	59 (41,8)	20 (14,2)
	K	0,65	0,89	4 (2,8)	8 (5,7)	41 (29,1)	69 (48,9)	19 (13,5)
**06 Ängstlichkeit / Unsicherheit / Traurigkeit**	E	0,52	0,78	0 (0,0)	8 (5,7)	68 (48,2)	48 (34,0)	17 (12,1)
	K	0,57	0,94	3 (2,1)	9 (6,4)	60 (42,6)	42 (29,8)	27 (19,1)
**07 Veränderungen für Kind insgesamt**	E	0,84	0,92	1 (0,7)	16 (11,3)	19 (13,5)	74 (52,5)	31 (22,0)
	K	0,71	0,96	3 (2,1)	14 (9,9)	31 (22,0)	66 (46,8)	27 (19,1)
**CBB-Gesamtbelastungsindex**	E (C)	0,65	0,56					
	K	0,61	0,54					

Anmerkungen. n = absolute Häufigkeiten, *%* = prozentuale Häufigkeiten, *M* = Mittelwert, *SD* = Standardabweichung, *E* = Eltern, *K* = Kind, *E(C)* = CBB-E-Cross-Informant-Skala.

Onlinematerial 5: Stärke der Belastungen durch die Corona-Pandemie in der *(Vor-)Schulstichprobe* (Eltern- und Selbsturteil vorliegend; *n* = 672), Angaben auf Item-Ebene

	Urteil	*M*	*SD*	***n (%)***				
				**viel besser (-2)**	**etwas besser (-1)**	**unverändert (0)**	**etwas schlechter (1)**	**viel schlechter (2)**
**01 Beziehungen Kind zu Familienmitgliedern**	E	-0,05	0,65	14 (2,1)	109 (16,2)	452 (67,3)	89 (13,2)	8 (1,2)
	K	0,03	0,66	16 (2.4)	78 (11,7)	453 (87,7)	114 (17,0)	8 (1,2)
**02 Beziehungen Kind zu Freund:innen**	E	0,39	0,76	6 (0,9)	48 (7,2)	343 (51,2)	226 (33,7)	47 (7,0)
	K	0,31	0,88	23 (3,4)	82 (12,2)	267 (39,7)	261 (38,8)	39 (5,8)
**03 Belastungen durch Schule / Lernen**	E	0,54	0,91	8 (1,2)	81 (12,1)	206 (30,9)	284 (42,6)	88 (13,2)
	K	0,44	0,93	16 (2,4)	82 (12,2)	236 (35,2)	262 (39,0)	75 (11,2)
**04 Freizeit / Beschäftigungsmöglichkeit**	E	1,47	0,75	3 (0,4)	9 (1,3)	58 (8,6)	201 (29,9)	401 (59,7)
	K	1,23	0,91	9 (1,3)	28 (4,2)	78 (11,6)	240 (35,7)	317 (47,2)
**05 Gereiztheit / Unruhe / Unausgeglichenheit**	E	0,48	0,76	6 (0,9)	42 (6,3)	297 (44,5)	271 (40,6)	51 (7,6)
	K	0,44	0,74	5 (0,7)	40 (6,0)	322 (48,1)	258 (38,5)	45 (6,7)
**06 Ängstlichkeit / Unsicherheit / Traurigkeit**	E	0,33	0,64	6 (0,9)	24 (3,6)	403 (60,1)	215 (32,1)	22 (3,3)
	K	0,29	0,71	11 (1,6)	37 (5,5)	405 (60,3)	187 (27,8)	32 (4,8)
**07 Veränderungen für Kind insgesamt**	E	0,87	0,73	-	27 (4,0)	146 (21,7)	387 (57,6)	112 (16,7)
	K	0,52	0,86	15 (2,2)	72 (10,7)	179 (26,7)	356 (53,1)	48 (7,2)
**CBB-Gesamtbelastungsindex**	E (C)	0,58	0,46					
	K	0,47	0,47					

Anmerkungen. n = absolute Häufigkeiten, *%* = prozentuale Häufigkeiten, *M* = Mittelwert, *SD* = Standardabweichung, *E* = Eltern, *K* = Kind, *E(C)* = CBB-E-Cross-Informant-Skala.

Onlinematerial 6: Signifikante Korrelationen mit korrespondierendem CBB-Gesamtindizes bzw. Cross-informant-Index

Skala<br>(CBCL: *n* = 254; YSR: *n* = 141)	Signifikante Korrelation mit korrespondierenden CBB-Gesamtindizes	Signifikante Korrelation mit korrespondierenden CBB-Cross-Informant-Index
CBCL - Gesamt	.20*	.24**
CBCL - Externale Probleme	-	-
CBCL - Internale Probleme	-	-
CBCL - Ängstlich/depressiv	.21**	.25**
CBCL - Rückzügig/depressiv	-	.18**
CBCL - Körperliche Beschwerden	-	.14*
CBCL - Soziale Probleme	-	-
CBCL - Denk-, (Schlaf-) & repetitive Probleme	.19**	.18**
CBCL - Aufmerksamkeitsprobleme	-	-
CBCL - Regelverletzendes Verhalten	-	-
CBCL - Aggressives Verhalten	.13*	.14*
YSR - Gesamt	.25**	
YSR - Externale Probleme	.22**..	
YSR - Internale Probleme	.25**..	
YSR - Ängstlich/depressiv	.25**	
YSR - Rückzügig/depressiv	-	
YSR - Körperliche Beschwerden	.26**	
YSR - Soziale Probleme	-	
YSR - Denk-, (Schlaf-) & repetitive Probleme	.24**	
YSR - Aufmerksamkeitsprobleme	-	
YSR - Regelverletzendes Verhalten	.22**	
YSR - Aggressives Verhalten	.19*	

Anmerkungen. CBB = Corona-Belastungsbogen*, CBCL* = Child Behavior Checklist*, YSR* = Youth Self Report, *n* = absolute Häufigkeiten.
